# HOPE survival probability cutoff for ECLS rewarming in hypothermic cardiac arrest

**DOI:** 10.1016/j.resplu.2024.100616

**Published:** 2024-03-23

**Authors:** Valentin Rousson, Nicolas Hall, Mathieu Pasquier

**Affiliations:** a Center for Primary Care and Public Health (Unisanté), Lausanne, Switzerland; b Department of Emergency Medicine, Lausanne University Hospital, Lausanne, Switzerland; c Department of Emergency Medicine, Lausanne University Hospital and University of Lausanne, Switzerland

**Keywords:** Accidental hypothermia, Cardiac arrest, Cutoff, Decision making, ECLS rewarming, False negative: False positive, HOPE score, Survival probability

## Abstract

The HOPE score (https://www.hypothermiascore.org) is a validated instrument for estimating the survival probability of patients in hypothermic cardiac arrest with ECLS rewarming. It is based on six patient characteristics: sex, age, mechanism of hypothermia, duration of cardiopulmonary resuscitation, serum potassium and temperature. The HOPE score provides a reliable estimate of survival probability that can be used to decide whether to rewarm a patient. In the initial publication of the HOPE score, a cutoff of 10% was proposed, below which a patient would not be rewarmed. This choice was tentative and subject to debate. In this paper, we examine the implications of this choice on the proportions of false positives (i.e., rewarmed patient who ends up dying) and false negatives (i.e., non-rewarmed patients who would have survived if rewarmed), and we provide approximate formulas to obtain upper bounds for these proportions as a function of the cutoff chosen. In particular, the choice of a 10% cutoff will result in a proportion of FP of less than 40% and a proportion of FN of less than 0.5% in many practical situations.

## Introduction

Accidental hypothermia is a potentially reversible cause of cardiac arrest (CA).^1^ The treatment of choice for patients in hypothermic CA is extracorporeal life support (ECLS) rewarming. The in-hospital decision to use ECLS was traditionally based on serum potassium, with values lower than a certain cutoff used to decide whether ECLS rewarming was indicated.^1^, ^2^, ^3^

This historical approach changed with the 2021 European resuscitation council (ERC) guidelines that advise the use of multivariable triage tools, such as the ICE score and the HOPE score rather than single potassium levels.^4^, ^5^, ^6^ The HOPE score is a continuous, rather than discrete, instrument for estimating the probability of survival of patients undergoing ECLS rewarming and can therefore guide clinical decisions to rewarm or not hypothermic CA patients. The HOPE score has been externally validated.^4^, ^5^, ^7^

In the initial publication of the HOPE score, a cutoff of 10% was proposed, below which a patient would not be rewarmed. However, the advantage of having a continuous and accurate survival probability is to leave open the possibility of using a different cutoff, depending on time, location and possibly the subpopulation in which it is used, e.g. a population of children.^7^ It therefore seems important to understand the implications of the cutoff choice in terms of proportions of false positives and false negatives, which we will examine in this short article.

## The HOPE score

The HOPE (for Hypothermia Outcome Prediction after ECLS) score estimates the probability of survival at hospital discharge for hypothermic CA patients undergoing ECLS rewarming. It was derived from a retrospective study of 286 hypothermic CA patients rewarmed with ECLS and validated using data from another 122 corresponding patients. It achieved an estimated AUC of 0.89, or 0.87 when corrected for optimism, in the derivation sample and 0.83 in the validation sample.^7^ In comparison, the AUC for a triage tool based solely on the potassium value would lie below 0.8. As a measure of discrimination, AUC values between 0.8 and 0.9 were considered “excellent”, and those between 0.7 and 0.8 “acceptable”.^8^

The statistical model defining the HOPE score was derived using logistic regression and included the six following predictors: sex (female or male); age; mechanism of hypothermia (non-asphyxia-related or asphyxia-related); duration of cardiopulmonary resuscitation; serum potassium level at admission; and core temperature at admission.^5^ From there, an equation for calculating the survival probability of a patient undergoing ECLS rewarming (i.e., a number between 0% and 100%) is available. Recall that the inverse of such a survival probability provides a “number needed to treat” to save one patient. For example, a survival probability of 10% indicates that1/0.1 = 10 patients need to be rewarmed to expect to save one life. The website to calculate HOPE can be accessed via: https://www.hypothermiascore.org.

## False positives and false negatives

In the HOPE validation study, both calibration and discrimination of the HOPE score were found to be good, meaning respectively that the HOPE survival probability is considered reliable and that it is effective in discriminating survivors from non-survivors. Its use can help to decide not to rewarm a patient if the survival probability is deemed to be too low. In this case, a practical (and ethical) question is to define what should be considered a low probability of survival, i.e., how to dichotomise the HOPE survival probability. A 10% survival probability cutoff was used in the initial publication of the HOPE score but opinions on this may change depending on what subjectively appears to be a sufficiently high probability of survival.

As in all dichotomous decisions based on an arbitrary cutoff, there is a trade-off between the “false positive” (FP) and “false negative” (FN) decisions. By lowering the cutoff, there will be more FP and fewer FN, and the opposite by raising it. In our case, a FP corresponds to a rewarmed patient who ends up dying and a FN would be a non-rewarmed patient who would have survived if rewarmed. The cost of a FN is clearly higher, which is why we advocate using a low cutoff such as 10% (or even less) when dichotomising the HOPE survival probability. An important question is how many FP and FN are expected depending on the cutoff used.

Table 1 shows the numbers and percentages of true positives (TP), true negatives (TN), false positives (FP) and false negatives (FN) for the 286 patients in the HOPE derivation study and the 122 patients in the HOPE validation study, when a cutoff of 10% was used to decide whether to rewarm a patient. The proportions of FN were low, 0% in the derivation study and less than 1% in the validation study, while the proportions of FP were respectively 31% and 30%.

**Table 1:** Numbers and percentages of true positives (TP), true negatives (TN), false positives (FP) and false negatives (FN) using a cutoff of 10% to decide whether to rewarm a patient, for the286 patients in the HOPE derivation study (left part of the table), and the 122 patients in the HOPE validation study (right part of the table).

**DERIVATION**	Survived	Not survived	**VALIDATION**	Survived	Not Survived
Rewarmed	**106 (TP = 37%)**	**88 (FP = 31%)**	Rewarmed	**50 (TP = 41%)**	**37 (FP = 30%)**
Not rewarmed	**0 (FN = 0%)**	**92 (TN = 32%)**	Not rewarmed	**1 (FN = 1%)**	**34 (TN = 28%)**

It is however important to recall that the proportions of FP and FN depend not only on the cutoff used but also on the patients’ characteristics in a given study. As a first example, let’s consider an idealized sample of 100 patients where the HOPE survival probability is either 40% (for 50 patients), or just below 10% (for the remaining 50 patients). This means that with a cutoff of 10%, only the former 50 patients will be rewarmed. As the HOPE survival probabilities are well calibrated, we expect in this case 0.4x50 = 20 survivors (and therefore 30 non-survivors, i.e. 30 FP) among the former 50 patients, and 0.1x50 = 5 survivors (i.e. 5 FN) among the latter, and thus 30/100 = 30% of FP and 5/100 = 5% of FN. If the HOPE survival probabilities were of 80% for the former 50 patients, and 2% for the latter, we would expect 0.8x50 = 40 survivors (and therefore 10 FP) among the former 50 patients, and 0.02x50 = 1 survivor (1 FN) among the latter. This means 10/100 = 10% of FP and 1/100 = 1% of FN, which is different from the first example, although the same cutoff is used.

In terms of FN, the theoretical worse-case scenario is a situation in which 100% of the patients would have a HOPE survival probability just equal to (or slightly below) the 10% cutoff. In this case, the proportion of FN would be precisely equal to or slightly below 10%.

## Practical upper bounds

While we just saw that the survival probability cutoff below which a patient would not be rewarmed can be interpreted as the maximum proportion of FN that one would be willing to accept, the actual proportion of FN will be much lower in most realistic situations, as seen in the HOPE derivation and validation studies. In this section, we provide useful approximations for the proportions of FP and FN that can be achieved in practice, depending on the cutoff used.

Table 2 provides the proportions of TP, TN, FP and FN achieved using a cutoff C, when the survival probabilities are well calibrated and uniformly distributed between 0% and 100%. The proportions of FP and FN are given respectively by (1-C)^2^/2 and C^2^/2. One can therefore easily calculate these proportions depending on the cutoff used, obtaining for example FP = 40.5% and FN = 0.5% for a cutoff C = 10%.

**Table 2:** Percentages of true positives (TP), true negatives (TN), false positives (FP) and false negatives (FN) using a cutoff of C to decide whether to rewarm a patient, when assuming well calibrated and uniformly distributed survival probabilities between 0% and 100*%.*

	Survived	Not survived
Rewarmed	**TP=(1-C^2^)/2**	**FP=(1-C)^2^/2**
Not rewarmed	**FN = C^2^/2**	**TN = C(1-C/2)**

**Fig. 1:**
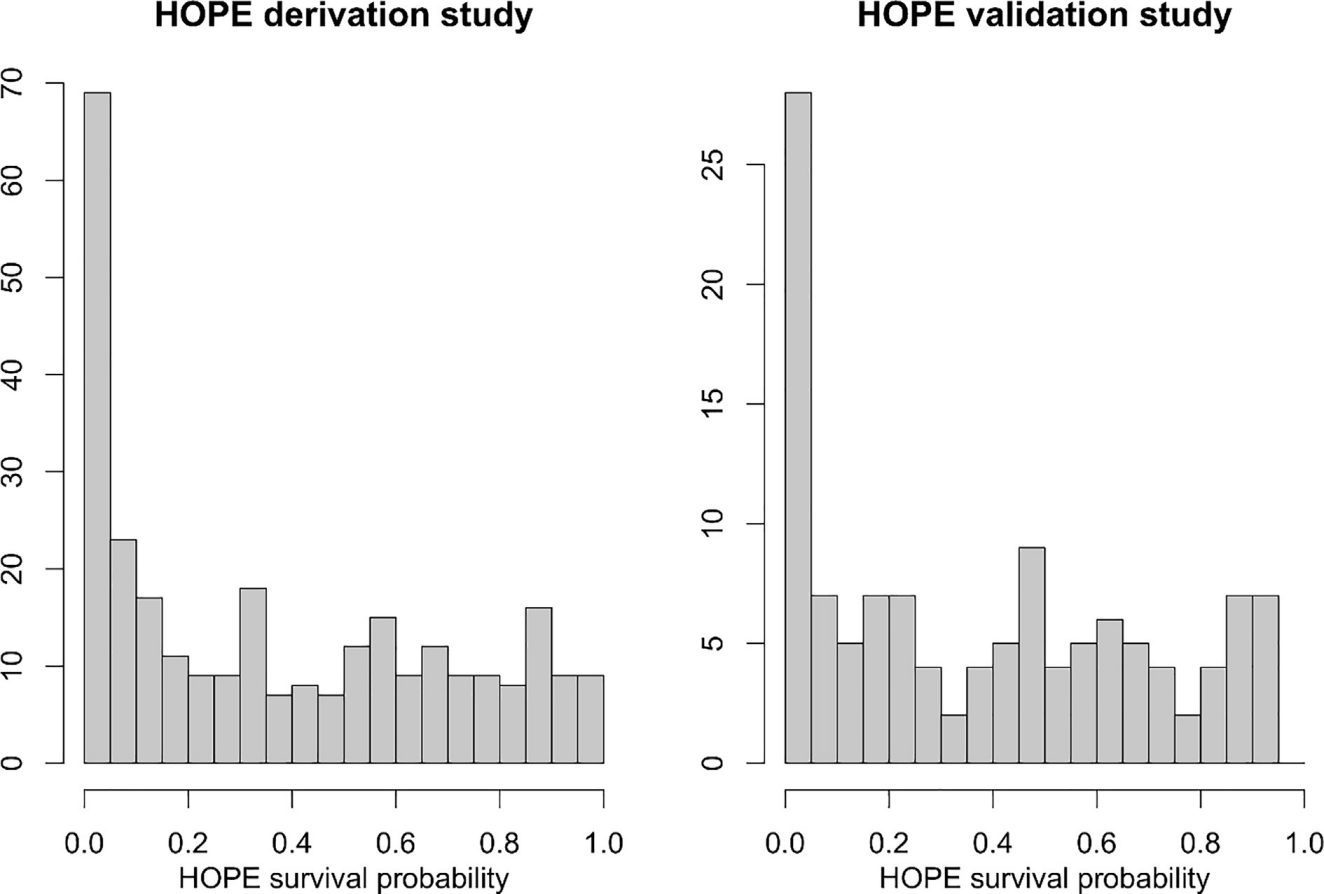
Distribution of HOPE survival probabilities for the 286 patients in the HOPE derivation study (left panel), and for the 122 patients in the HOPE validation study (right panel).

Of course, a uniform distribution of survival probabilities may not be realistic, but it could represent a practical worse-case scenario. Fig. 1 shows the distribution of the HOPE survival probabilities in the HOPE derivation and validation studies. There is a clear excess of small survival probabilities compared to a uniform distribution. However, the rest of the distribution looks roughly uniform. This means that a realistic model for the distribution of survival probabilities could be a mixture of a uniform distribution with a proportion Z of near-zero probabilities. An excess of near-zero probabilities will in fact reduce the proportions of FP and FN by a factor close to (1-Z) compared with a uniform distribution. Thus, the formulas provided in Table 2 can be interpreted as upper bounds for FP and FN proportions, applicable in many practical cases. For example, with a proportion Z = 20% of near-zero probabilities, we would reach proportions of FP = 0.8x40.5 = 32.4% and FN = 0.8x0.05 = 0.4%, which are close to the proportions of FP and FN given in Table 1.

## Conclusions

The HOPE score has replaced the use of potassium values alone to decide whether to rewarm patients with hypothermic CA. It can be reliably interpreted as a probability of survival for a patient undergoing ECLS rewarming and used to guide clinical decisions whether to rewarm a patient or not, based on a certain cutoff. In this article, we have provided simple formulas for the upper bounds of FP and FN proportions in function of this cutoff. This should help clinicians or inspire guidelines for choosing a cutoff, based on the proportions of FP and FN that one is willing to accept. In particular, the choice of a cutoff of 10%, as was proposed, implies a proportion of FP of less than about 40%, and a proportion of FN of less than 0.5%, which may still appear a good compromise.

## Funding

This research received no external funding. The article processing charges were funded by the Lausanne University Open Access program.

## CRediT authorship contribution statement

**Valentin Rousson:** Writing – review & editing, Writing – original draft, Validation, Methodology, Conceptualization. **Nicolas Hall:** Writing – review & editing, Writing – original draft, Validation. **Mathieu Pasquier:** Writing – review & editing, Writing – original draft, Validation, Methodology, Conceptualization.

## Declaration of competing interest

The authors declare that they have no known competing financial interests or personal relationships that could have appeared to influence the work reported in this paper.
